# Cytotoxic, anti-proliferative, and apoptotic evaluation of *Ramalina sinensis* (*Ascomycota, Lecanoromycetes*), lichenized fungus on oral squamous cell carcinoma cell line; in-vitro study

**DOI:** 10.1186/s12906-023-04118-1

**Published:** 2023-08-22

**Authors:** Maryam Koopaie, Hanieh Karimi, Mohammad Sohrabi, Hooman Norouzi

**Affiliations:** 1 Department of Oral Medicine, School of Dentistry, Tehran University of Medical Sciences, North Kargar St, P.O. Box: 14395 -433, Tehran, 14399-55991 Iran; 2 Department of Biotechnology, Iranian Research Organization for Science and Technology, Tehran, Iran; 3 Former graduate student of the Department of Horticultural Sciences, Faculty of Agriculture, Bu-Ali Sina University, Hamedan, Iran

**Keywords:** Oral squamous cell carcinoma (OSCC), Cell viability, Apoptosis, *Ramalina sinensis*

## Abstract

**Background:**

Scientists and medical professionals are actively striving to improve the efficacy of treatment methods for oral squamous cell carcinoma (OSCC), the most frequently occurring cancer within the oral cavity, by exploring the potential of natural products**.** The active pharmacological compounds found in lichenized fungi have shown potential for aiding in cancer treatment. Recent research aims to evaluate the impact of the lichenized fungus *Ramalina sinensis* (*R. sinensis*) on the cell viability and apoptosis of OSCC cell lines, considering the anti-inflammatory and anti-cancer capabilities of lichens.

**Methods:**

*Ramalina sinensis (Ascomycota, Lecanoromycetes)* was selected for investigation of its effects on a human oral squamous cell carcinoma cell line. Acetone and methanol extracts of *R. sinensis* on an OSCC cell line (KB cell line, NCBI Code: C152) were investigated. Viability was assessed by MTT assay analysis, and apoptotic cells were measured using flow cytometry analysis. Scratch assay was used to assess cell migration. The chemical composition and metabolic profiling of *R. sinensis* were investigated.

**Results:**

The growth and multiplication of KB cells were observed to undergo a gradual but remarkable inhibition when exposed to various concentrations. Specifically, concentrations of 6.25, 12.5, 25, 50, 100, and 200 μg/mL exhibited a significant suppressive effect on the proliferation of KB cells. The inhibition of cell proliferation exhibited a statistically significant difference between the extracts obtained from acetone and methanol. Flow cytometry results show an increase in apoptosis of OSCC cells by acetone extract. *R. sinensis* exerted a concentration-dependent inhibitory effect on the migration of OSCC cells. The chemical composition of *R. sinensis* was investigated using liquid chromatography positive ion electrospray ionization tandem mass spectrometry (LC–ESI–MS/MS), and 33 compounds in the acetone and methanol extracts of *R. sinensis* were detected.

**Conclusion:**

The findings provide evidence supporting the beneficial effects of *R. sinensis* extract on inducing apoptosis in OSCC cells and exerting anti-cancer properties.

**Supplementary Information:**

The online version contains supplementary material available at 10.1186/s12906-023-04118-1.

## Background

Oral squamous cell carcinoma (OSCC) is the most prevalent form of oral cancer, accounting for approximately 90% of all oral malignancies [1]. Oral squamous cell carcinoma (OSCC) is a multifactorial disease with various extrinsic and intrinsic factors contributing to its incidence and progression, including smoking, alcohol intake, iron deficiency anemia, and genetics [2]. While surgery, radiotherapy, chemotherapy, or a combination of these methods are commonly used treatment approaches [3]. Despite advancements in treatment modalities, the survival rate of oral cancer remains low, and attempts are underway to provide novel and effective treatment modalities considering the dismal prognosis of oral cancer [4].

Lichens, which are a source of active pharmacological compounds, have recently gained attention for their potential use in treatment purposes [5, 6].

Lichen metabolites are categorized into primary and secondary metabolites. Primary metabolites, such as proteins, lipids, and carbohydrates, contribute to the structure and metabolism of lichens. In contrast, secondary metabolites are complex compounds that possess therapeutic properties and potential uses [7]. Derived from lichens, over 1000 metabolites have been identified, which exhibit a wide range of biological activities, including antioxidant, cytotoxic, anti-inflammatory, and anti-proliferative properties [8]. Bioactive metabolites, such as the phenolic chemicals despides, depsidones, and dibenzofurans, are produced by lichens, which are symbiotic organisms composed of a fungus and an algae/cyanobacteria [8].

Numerous lichen species have been identified as having traditional medicinal uses [9]. Their traditional usage dates back to Ancient Greece when one of its species was used to treat inflammation, jaundice, and impetigo. *Ramalina calicaris, R. conduplicans, R. farinacea, R. inflata, R. menziesii, R. roesleri,* and *R. sinensis* are some of the Ramalina species that have been used in traditional medicine [10, 11].

Furthermore, a growing body of evidence suggests that various lichen substances can interact with multiple molecular mechanisms that play crucial roles in the regulation of cell death, including cell cycle arrest, apoptosis, necrosis, and inhibition of angiogenesis [12]. The mechanisms involved in cell death caused by lichens are complex and may involve several processes, including cell cycle arrest, apoptosis, necrosis, and inhibition of angiogenesis [13]. Programmed cell death (PCD) is another active cell death process that is regulated physiologically by a complex genetic mechanism. Some morphological features of PCD are common in fungi, plants, and animals, suggesting that this process is evolutionarily conserved. Gyrophoric acid, a lichen-derived metabolite, is known to trigger programmed cell death, and apoptosis of cells damaged by this compound occurs concomitant with changes in cell signaling pathways [14]. In another study, the mechanisms of cytotoxicity of four lichen secondary metabolites (parietin, atranorin, usnic acid, and gyrophoric acid) were investigated, and the results showed that these compounds induce apoptosis in human cancer cell lines [15]. Overall, the mechanisms involved in cell death caused by lichens are still not fully understood, and further research is needed to elucidate these mechanisms.

Anti-proliferative impact of three substances derived from lichen, namely protolichesterinic acid, was examined on human cancer cell lines by Haraldsdóttir et al. Their findings revealed that these substances exhibited significant anti-cancer effects [16]. Moreira et al. discussed the potential of *Ramalina lichenized fungi* to produce bioactive molecules that can be used as a model for the production of pharmaceuticals. They cited approximately 118 species of the *Ramalina genus* with published chemical or biological activity studies of extracts or isolated compounds. Extracts of several species presented significant results in biological tests, demonstrating the potential of these organisms to produce bioactive molecules that can be used as a model for the production of pharmaceuticals [17]. Shu et al. investigated the effect of Ramalin, a secondary metabolite from the Antarctic lichen *Ramalina terebrata*, on colorectal cancer cells. The results showed that Ramalin displayed concentration-dependent anti-cancer activity against HCT116 cells, significantly suppressing proliferation and inducing apoptosis [7]. Another study evaluated lichen extracts' antioxidant capacities, phenolic profiles, and cytotoxic effects. The results showed that some lichen extracts exhibited high cytotoxic effects against cancer cell growth [18].

Overall, these studies suggest that *Ramalina sinensis* lichenized fungus have the potential to be used as a source of bioactive compounds for the treatment of cancer. The aim of this study is to investigate the potential anti-cancer properties of Ramalina sinensis on oral squamous cell carcinoma (OSCC) cells. Specifically, the study evaluates the effects of Ramalina sinensis on cell viability and apoptosis in the OSCC cell line.

## Methods

### Lichen material

**Fig. 1:**
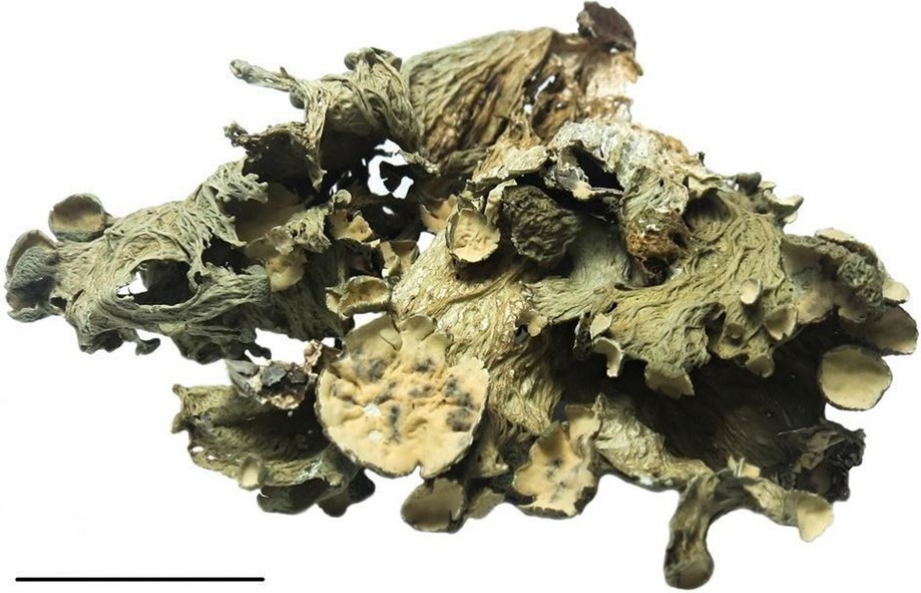
Thallus of *R. sinensis* (scale bar represents 10 mm)

In May 2018, several *Ramalina* specimens were collected from the Hyrcanian forest located in the Mazandaran Province of Iran, and *Ramalina sinensis (Ascomycota, Lecanoromycetes)* was chosen as the focus of this study. Lichen materials were collected outside of the protected area in accordance with the relevant guidelines and regulations of the Plant Varieties Protection, Environmental Protection Organization of Iran. The Museum of Iranian Lichens in the Iranian Research Organization for Science and Technology (IROST) (Tehran, Iran) provided the principal keys to identifying species (Fig. 1). The third author identified the lichen specimens, and a voucher specimen was deposited at the Museum of Iranian Lichens, located at the IROST in Iran (https://irost.org/museum/). After collecting the lichen specimens and ensuring that they were clean and free from debris, they were dried. After the lichens were fully dried, they were ground into a fine powder using a grinder to facilitate the extraction of the desired compounds. To extract the desired compounds, the cleaned lichen thallus was ground using a mortar and pestle under a small amount of liquid nitrogen, and acetone and methanol were used as separate solvents. The maceration method was employed, whereby 4 g of the powdered lichen material was dissolved in 100 ml of ethanol. The lichen material was then placed in a container with a tightly sealed lid, and the solvent, either acetone or methanol, was added to ensure full immersion. The mixture was soaked for 24 h, and the liquid containing the extracted compounds was filtered from the solid residue using a filtration setup with a 0.2 µm filter to sterilize it through injection. To increase the concentration of the extracted compounds, excess solvent was removed using vacuum distillation. Finally, the concentrated lichen extract was stored in amber glass vials at -80 °C in darkness to protect it from light degradation.. The cleaned lichen thallus was ground to a fine powder using a mortar and pestle under a small amount of liquid nitrogen.

### Cell lines and culture

The human oral epidermal carcinoma cell line (KB cell line, NCBI Code: C152) was obtained from the Pasteur Institute of Iran (Tehran, Iran) (Supplementary File). Cancer cell lines were cultured in Dulbecco’s modified eagle medium (DMEM) with 10% fetal bovine serum (FBS) and 1% PS at 37°C, 5.0% CO_2_, and 95.0% humidity. The necessary chemicals and reagents have been obtained from Merck Company (Merck, Germany).

### Cell viability

3-(4,5-dimethylthiazol-2-yl)-2,5-diphenyltetrazolium bromide (MTT) assay was used to determine viability, which is based on the reduction of MTT by mitochondrial dehydrogenase in intact cells into an insoluble purple formazan product [19]. In 96-well plates, cells (1*10^4^/well) were seeded, and after 24 h, the cells were treated with *R. sinensis* extract at various doses (200, 100, 50, 25, 12.5, and 6.25 μg/ml) and then *R. sinensis* -containing medium were carefully removed after the treatment. Cells were washed twice with phosphate-buffered saline (PBS) before each well received 100 mL media containing 0.5 mg/ml MTT in PBS, then the plate was incubated at 37°C for 4 h. The medium was then completely removed, 200 mL of tris (hydroxylmethyl) aminomethane (Tris)—dimethyl sulfoxide (DMSO) solution was added to each well, and the plate was vibrated for 30 min. Using an ELISA plate reader, the absorbance, as proportional to cell viability, was then measured in at least three independent measurements using a microplate reader (Bio-Rad Corp, California, USA) at 570 nm in each well and Gen 5 Version 2.07.17 software (BioTek, Winooski, USA) for data analysis. The average of measurements was reported for each well.

### Flow cytometry

Flow cytometry analysis was performed on apoptotic cells using a Beckman Coulter (EPICS XL). The Ramalina-treated and untreated cells were collected, twice-washed in PBS, fixed in 70.0% ethanol at 4°C for at least 12 h, centrifuged, and then incubated for 30 min at 37°C in the dark with 0.1% Triton X-100, 200 mg/ml RNase A, and 50 mg/ml propidium iodide (PI) in PBS. More than 3*10^4^/well cells were counted in each sample, and cells with lower DNA content than those in the G_0_/G_1_ phase were labeled as apoptotic [20, 21].

### Scratch assay

OSCC cells were cultured as confluent monolayers in the medium for 24 h. Cells across the well were removed using a standard 1400 μL pipette tip to induce wound formation. Wounded monolayers were then washed twice to remove non-adherent cells. The wound was observed with an inverted phase contrast microscope (Leica, Wetzlar, Germany) shortly after and again after 24 and 48 h. The experiment was conducted with a phase-contrast microscope and camera, capturing images of the scratch area at regular intervals (0, 24, and 48 h). Analysis of the migrated area involved importing the images into ImageJ software. The migration rate was estimated as the ratio of the mean distance between both borderlines generated by scratching cell-free after re-growing to the distance at t = 0 in the control and is represented as a percentage of the control. In quadruplicate, two separate sets of tests were carried out.

### Metabolic profiling of *R. sinensis*

The chemical composition of *R. sinensis* was investigated according to Norouzi et al. briefly [22]; lichen substances were separated by the Waters Alliance e2695 separation module (Milford, MA, USA) and the Atlantis T3 C18 column (2.1mm × 100 mm, 3 μm; Milford, MA, USA) and Column temperature set to 30℃. Samples were dissolved in methanol, and after filtration (PTFE membrane filters, 0.45 μm, Simplepure, China), 10 μL of each sample was injected into the separation module. Lichenochemicals were eluted within 25 min as follows: elution began by 95% (water (Milli-Q® integral water purification system (Merck, Germany)) + 0.1% formic acid (DiKMA Technologies, Beijing, China), v/v) and gradually decreased to 5% within 20 min. The Elution process went on for another 5 min with 95% acetonitrile (Thermo Fisher Scientific, Massachusetts, USA). The flow rate of eluents was adjusted to be 0.25 ml min^−1^. A Quattro micro API mass spectrometer (Milford, MA, USA) was used for tandem mass analysis. The MS/MS parameters applied were as follows: source temperature and desolvation temperature were set to be 120 ℃ and 300 ℃, respectively; capillary voltage, cone voltage, and collision energy were regulated at 3.5 kV, 30 V, and 30 eV, respectively; for both nebulizing and drying the gas, N_2_ was used. MassLynx 4.1 and MZmine 2.53 were used for data acquiring and analysis. Initial annotation of detected compounds was carried out based on the lichen spectral database (LDB) provided in the GNPS public spectral libraries [23]. Chemical structures were sketched using ChemDraw Ultra 12.0.

### Statistical analysis

SPSS software (version 22; SPSS Inc., Chicago, IL, USA) and GraphPad Prism 8.2.1 (GraphPad Software, San Diego, CA) were used for statistical analysis. All results are given as mean ± standard deviation (SD), with *p* ≤ 0.05 considered statistically significant.

## Results

**Fig. 2:**
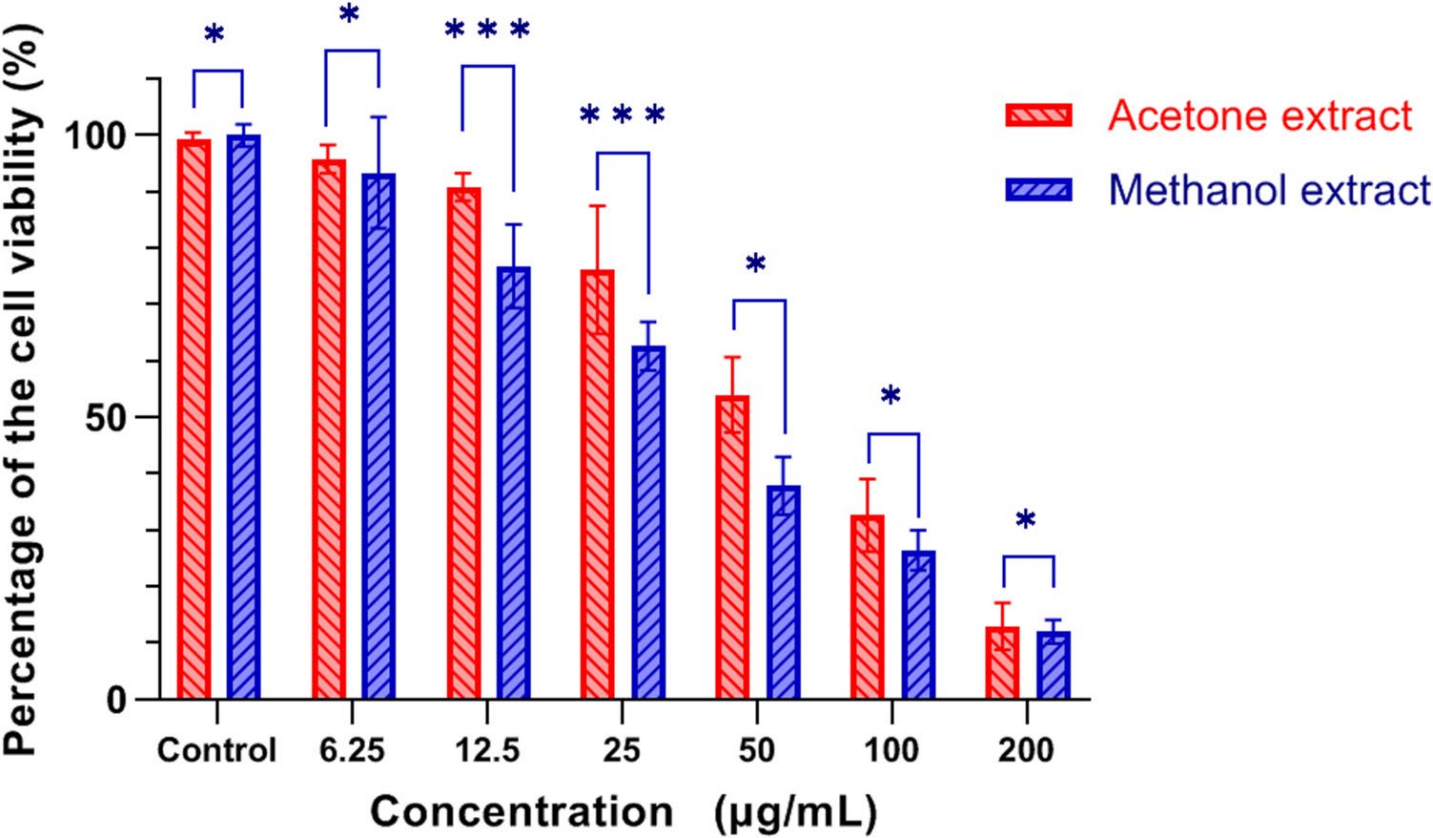
Percentage of cell viability (ratio of the treated group to control) after 24 h treatment. *: *P*-value (*p*) > 0.05, ***:* p* < 0.05

**Fig. 3:**
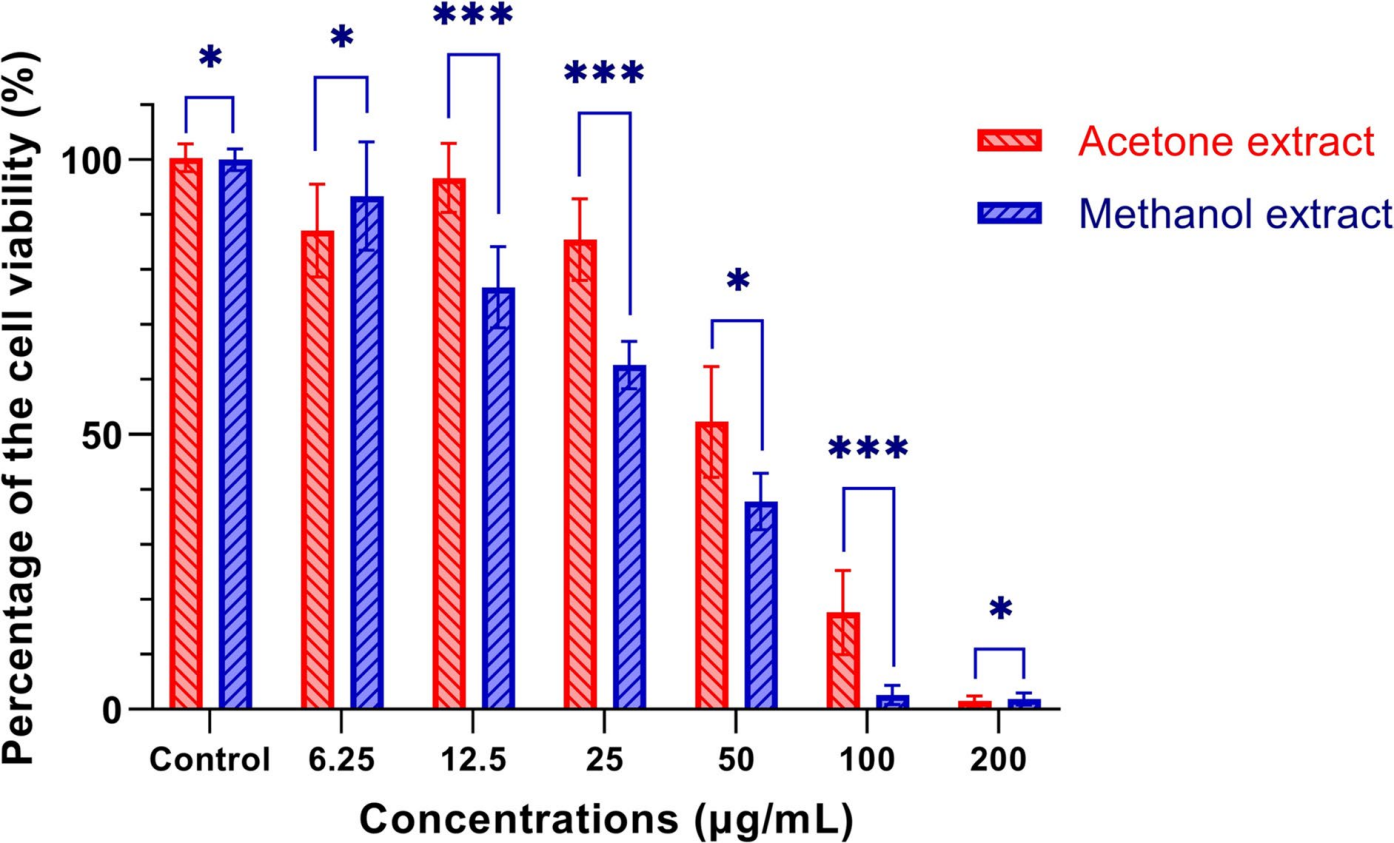
Percentage of cell viability (ratio of the treated group to control) after 48 h treatment. *: *p* > 0.05, ***: *p* < 0.05

**Fig. 4:**
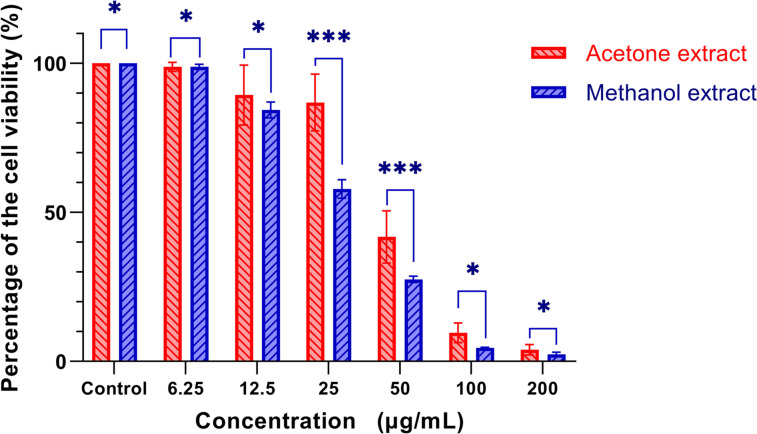
Percentage of cell viability (ratio of the treated group to control) after 72 h treatment. *: *p* > 0.05, ***: *p* < 0.05

To evaluate the effects of *R. sinensis* on the viability of oral squamous cell carcinoma (OSCC) cells, we performed an MTT assay using KB cells. The experiment involved treating the cells with different concentrations of *R. sinensis* (6.25, 12.5, 25, 50, 100, and 200 μg/mL) and incubating them for various durations (48 and 72 h). *R. sinensis* reduced the proliferation of KB cells in a time-dependent manner. The higher concentration (100 μg/mL) has a more considerable inhibitory impact than the lower 50 μg/mL concentration (Fig. 2 (cell viability after 24 h), (Fig. 3 (cell viability after 48 h), and Fig. 4 (cell viability after 72 h)).

The cell viability of OSCC cells treated with 100 μg/mL of acetone and methanol extract was 17.65% (SD =  ± 7.64%) and 2.60% (SD =  ± 1.77%), respectively. There was a statistically significant difference between acetone and methanol extract in the cell viability of OSCC cells treated with 100 μg/mL concentration (*p* = 0.029). The cell viability of OSCC cells at 200 μg/ml concentration was 1.48% (SD =  ± 0.92), and 1.89% (SD =  ± 1.09), for acetone and methanol extract, respectively, indicating a statistically significant difference (*p* < 0.05). The inhibitory concentration at 50% inhibition (IC50) was the parameter used to compare the cytotoxic activity, and a lower IC50 means better cytotoxic activity. The IC50 against KB cells was 50 μg/mL.

### Flow cytometry results

**Fig. 5:**
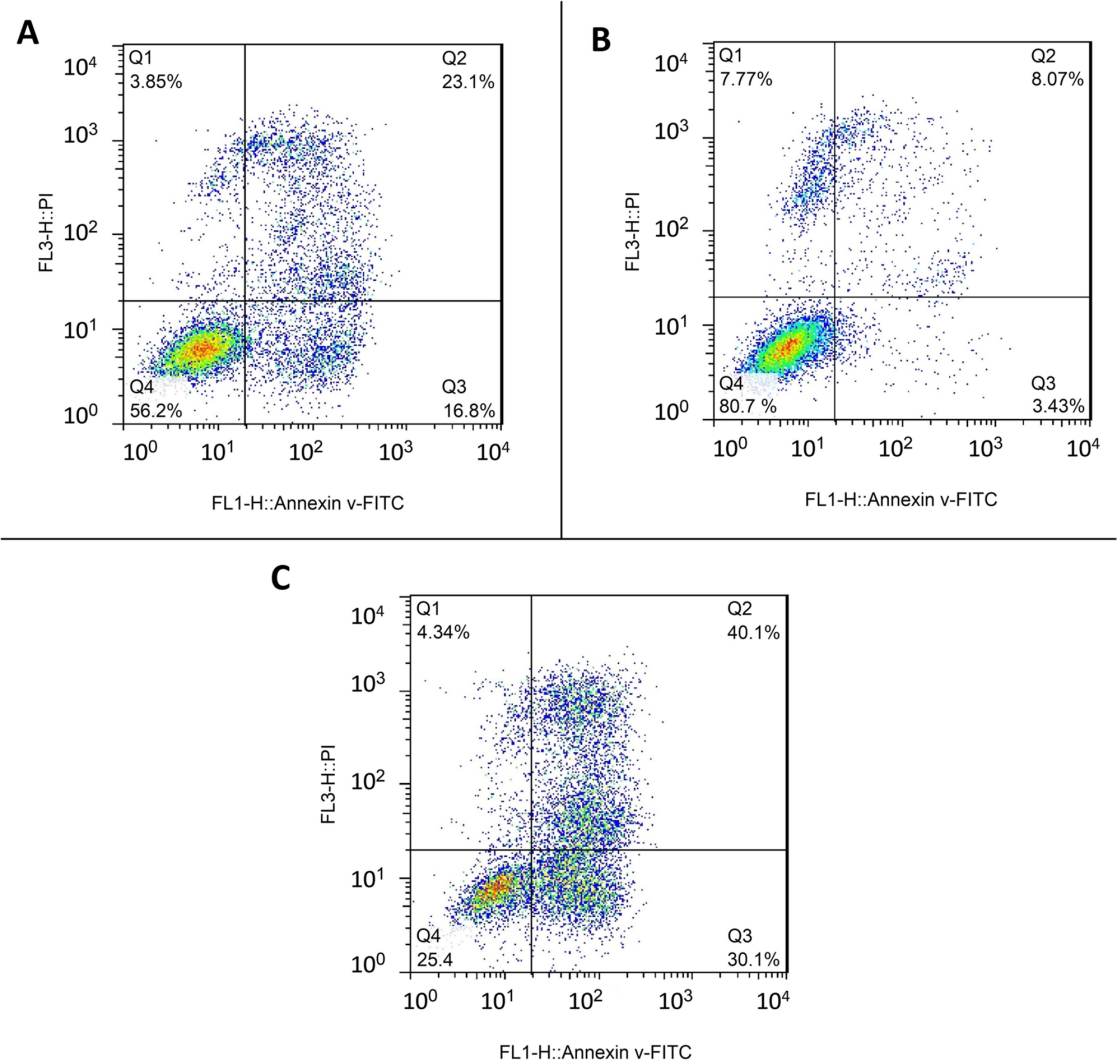
**A** Flow cytometry analysis after 24 h treated with 50 μg/mL of acetone extract of *R. sinensis*, **B** Flow cytometry analysis after 24 h in the control group, **C** Flow cytometry analysis after 24 h in the treated with 50 μg/mL of methanol extract of *R. sinensis*. Q1: Necrotic cells, Q2: Late Apoptotic cells, Q3: Early Apoptotic cells, Q4: Viable cells

The percent of OSCC cells *R. sinensis* treated 24 h (50 μg/mL of acetone extract of *R. sinensi*) in the early and late apoptosis phases were 16.8% and 23.1%, respectively (Fig. 5A). The percent of OSCC cells of control in the early apoptosis phase was 3.43% and 8.07% in the late apoptosis phase (Fig. 5B). Furthermore, the percent of *R. sinensis* treated (50 μg/mL of methanol extract) OSCC cells (24 h treatment) in the early and late apoptosis phase was 30.1% and 40.1%, respectively, which indicates an increase in apoptotic cells by methanol extract (Fig. 5C). The level of apoptosis, or programmed cell death, was significantly higher in the methanol extract compared to the acetone extract. In the methanol extract, early apoptosis accounted for 30%, and late apoptosis accounted for 40%, whereas in the acetone extract, early apoptosis accounted for 16.8%, and late apoptosis accounted for 23%.

These findings indicate that the methanol extract exhibited a higher apoptosis induction in cells than the acetone extract. Apoptosis is an important process in maintaining the balance between cell proliferation and cell death, and it can occur in response to various factors such as cellular stress, infection, and immune dysregulation. Understanding the level of apoptosis can be valuable in analyzing the effects of different extracts and identifying factors responsible for activating or inhibiting apoptosis in cells.

#### Scratch assay

**Fig. 6:**
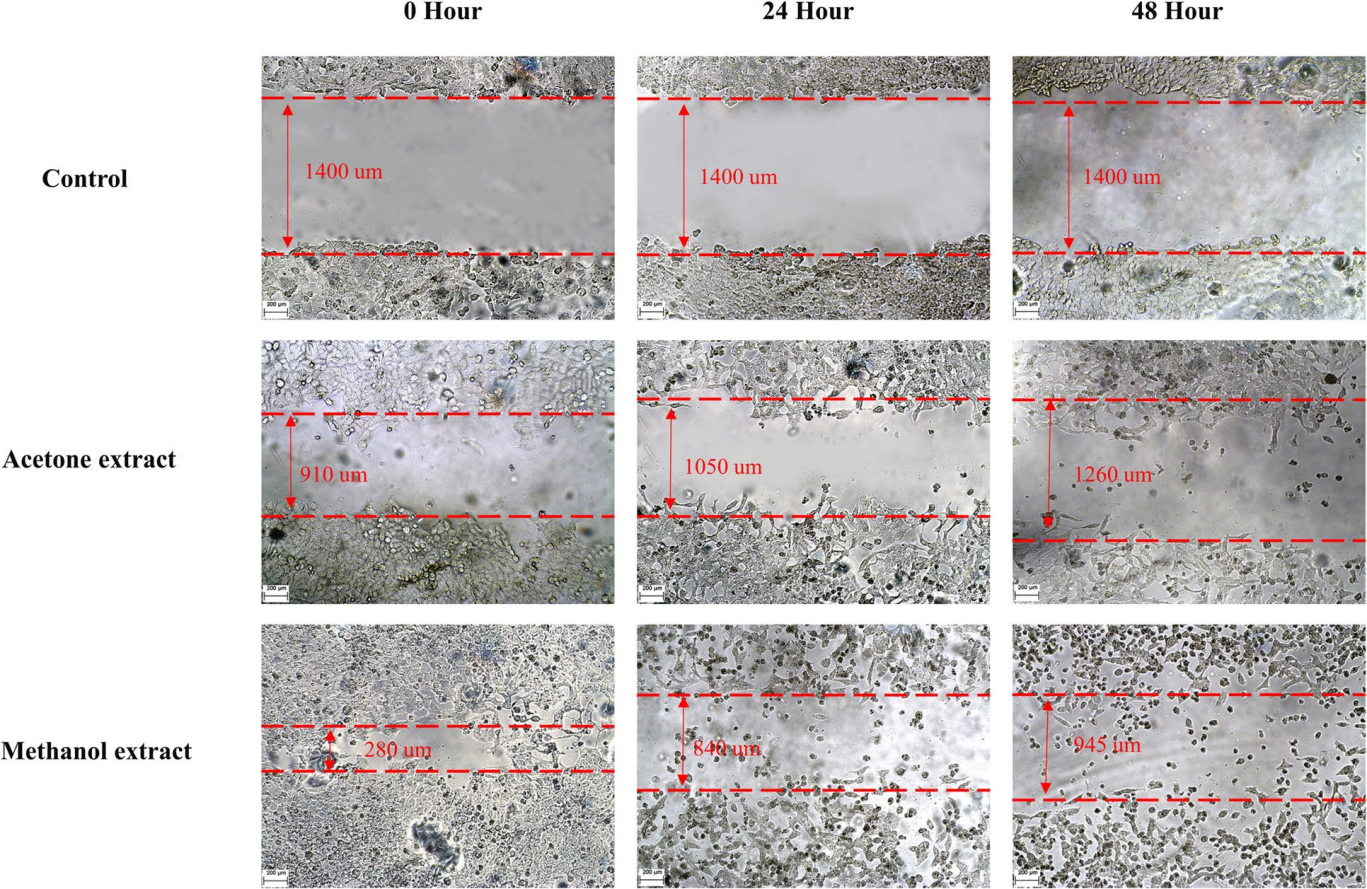
Scratch assay of OSCC cells in control, acetone extract, and methanol extract of *R. sinensis* at 0, 24, and 48 h of treatment

Non-cytotoxic doses of *R. sinensis* extract (50 μg/mL) were used to treat OSCC cells to assess the effect of *R. sinensis* on cell migration. *R. sinensis* extract significantly inhibited cell migration in OSCC cells (Fig. 6). In particular, the migration of cancer cells was inhibited by acetone and methanol extract of *R. sinensis.*

**Fig. 7:**
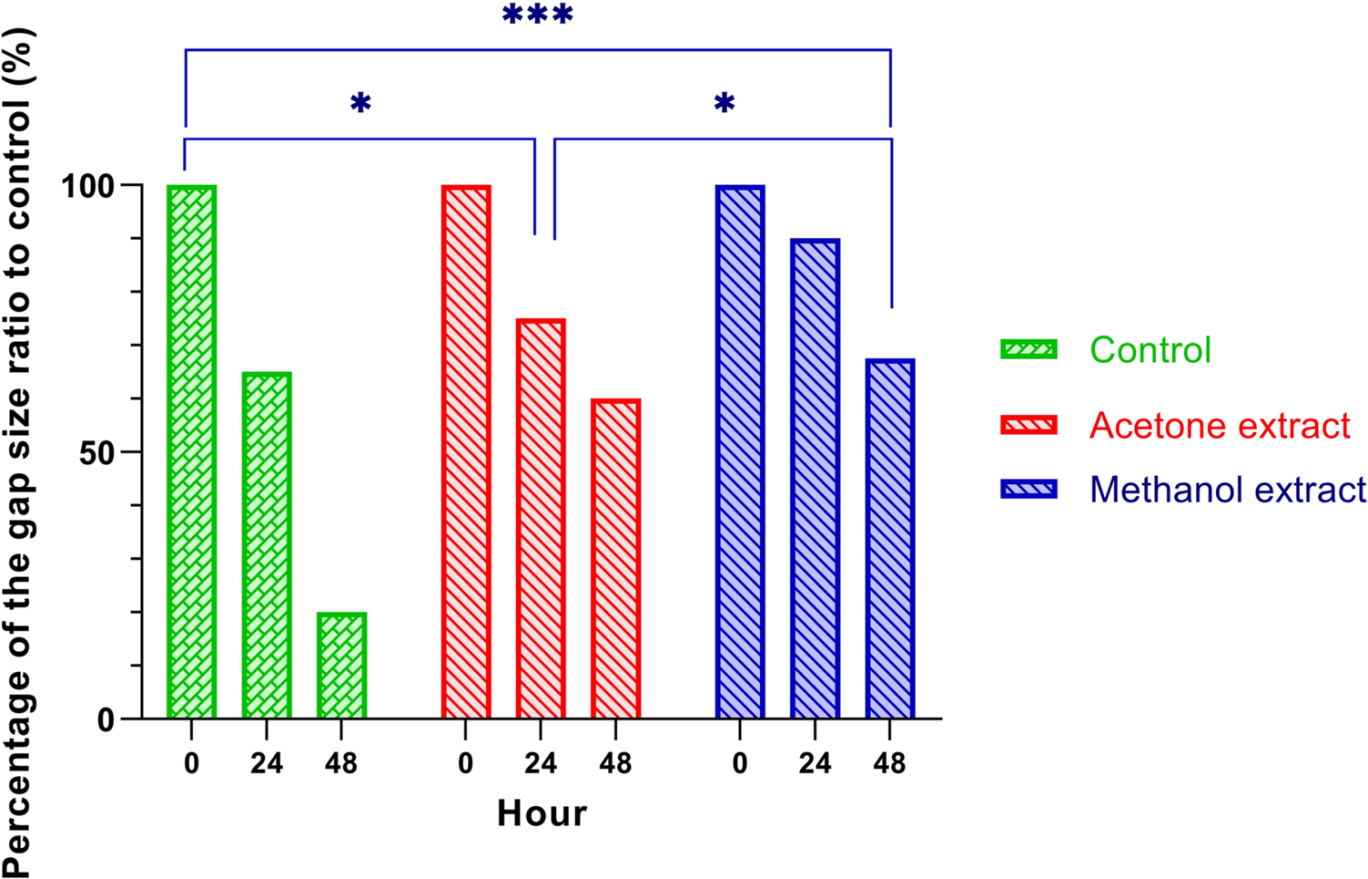
Scratch assay of OSCC cells in control, acetone extract, and methanol extract of *R. sinensis* at 0, 24, and 48 h of treatment. *: *p* > 0.05, ***: *p* < 0.05

These results indicate that *R. sinensis* suppresses the wound-healing process in human OSCC. There was no significant difference between acetone and methanol extract in cell migration inhibition. Therefore, *R. sinensis* may prevent cancer metastasis in OSCC cells (Fig. 7).

#### Metabolic profiling using LC–ESI–MS/MS

The chemical composition of *R. sinensis* was investigated using LC–ESI–MS/MS in negative ion mode (ESI^−^). We could detect 33 compounds in two extracts of *R. sinensis* that were mainly of chromones, depsides, dibenzofurans, depsidones, terpenoids, pulvinic acid derivatives, fatty acids, polyols, monocyclic aromatic compounds, along with small numbers of unknown compounds (Table 1)*.* In this study, two chromones were detected at *m/z* 235 and 361 that were tentatively identified as 6-hydroxymethyl eugenitin and lepraric acid, respectively. Depsides included ramalinaic acid (*m/z* 359), subsekikaic acid (*m/z* 389), 2-O-Methyldivaricatic acid (*m/z* 401), stenosporic acid (*m/z* 415), perlatolic acid (*m/z* 443), 2'-O-Methylperlatolic acid (*m/z* 457), and squamatic acid (*m/z* 802). Four molecular ions were tentatively identified as dibenzofurans, including isousnic acid (*m/z* 342), usnic acid (*m/z* 343), placodiolic acid (*m/z* 375), and pseudoplacodiolic acid (*m/z* 774). Usnic acid also indicated a 2M-2H + Na adduct at *m/z* 710. Besides this, five depsidones were also identified as virensic acid, norstictic acid, conprotocetraric acid, methylstictic acid, and gangaleoidin at *m/z* 356, 371, 376, 399, and 412, respectively. Ceruchinol ((-)-ent-Kauran-16**α**-ol), a diterpene, was the only terpenoid identified at *m/z* 289. We could also identify two fatty acids: nephrosterinic acid (*m/z *295) and arachidonic acid (*m/z *304). Atranol and olivetolic acid are monocyclic aromatic compounds tentatively identified at *m/z *151 and 223, respectively. Meso-erythritol was the only polyol we could identify at *m/z* 121. Finally, a molecular ion at *m/z* 350 proved to be a pulvinic acid derivative, further identified as leprapinic acid. Moreover, this compound also showed an adduct (2M-2H + Na) at *m/z* 725.

**Table 1:** MS/MS-based characterization of *R. sinensis* compounds extracted with different solvents

**Tentative identification**	**Chemical formula of [M-H]**^**−**^	**R**_**t**_** (min)**	**Calculated mass**	**[M-H]**^**−**^ ***m/z***	**MS**^**2**^ **ions**	**Type of extract**		**Structure**
						**Acetone**	**Methanol**	
**Chromones**								
6-Hydroxymethyl eugenitin	C_12_H_11_O_5_	11.80	235.0606	235	235, 232, 220, 207, 190, 164, 148, 137, 83, 79, 73	+	-	
Lepraric acid	C_18_H_17_O_8_	13.10	361.0923	361	235, 220, 207, 191	+	-	
**Depsides**								
Ramalinaic acid	C_18_H_15_O_8_	11.90	359.0766	359	343, 326, 323, 301, 283, 275, 257, 229, 215, 83	+	-	
Subsekikaic acid	C_20_H_21_O_8_	15.16	389.1236	389	389, 343, 328, 325, 315, 301, 299, 286, 259, 231, 220, 180, 139, 95, 83	+	-	
2-O-Methyldivaricatic acid	C_22_H_25_O_7_	12.94	401.1600	401	356, 342, 325, 282, 270, 226, 218, 181, 96, 68	-	+	
Stenosporic acid	C_23_H_27_O_7_	13.54	415.1756	415	370, 355, 343, 338, 326, 314, 231, 160	+	+	
Perlatolic acid	C_25_H_31_O_7_	14.43	443.2069	443	398, 366, 357, 339, 326, 313, 297, 259, 258, 218	+	-	
2'-O-Methylperlatolic acid	C_26_H_33_O_7_	15.00	457.2226	457	412, 397, 386, 354, 340, 314, 297, 239, 214, 180, 94	-	+	
Squamatic acid	2 M-2H + Na	15.10	801.164	802	412, 391, 389	-	-	
**Tentative identification**	**Chemical formula of [M-H]**^**−**^	**R**_**t**_** (min)**	**Calculated mass**	**[M-H]**^**−**^ ***m/z***	**MS**^**2**^** ions,** ***m/z***	**Type of extract**		**Structure**
						**Acetone**	**Methanol**	
**Dibenzofurans**								
Isousnic acid		13.58		342	312, 286, 231, 82	+	-	
Usnic acid	C_18_H_15_O_7_	15.44	343.0817	343	328, 313, 299, 259, 231, 215, 189, 83	+	+	
Placodiolic acid	C_19_H_19_O_8_	14.47	375.1079	375	375, 373, 357, 343, 328, 315, 299, 255, 247, 234, 231, 215, 180, 83	+	-	
Usnic acid	2 M-2H + Na	15.42	709.153	710	343, 328	+	+	
Pseudoplacodiolic acid	2 M-2H + Na	14.51	773.206	774	774, 375, 233	-	+	
**Depsidones**								
Virensic acid		12.95		356	341, 326, 313, 311, 299, 297, 284, 272, 267, 243, 231, 177, 163, 96, 59	-	+	
Norstictic acid	C_18_H_11_O_9_	13.49	371.0403	371	369, 340, 338, 326, 323, 313, 309, 299, 231, 216, 207, 110, 79	-	+	
Conprotocetraric acid	C_18_H_16_O_9_	14.47	376.0794	376	343, 328, 315, 301, 299, 291, 286, 283, 271, 259, 256, 244, 231, 227, 196, 187, 180, 95, 82, 55	-	+	
**Tentative identification**	**Chemical formula of [M-H]**^**−**^	**R**_**t**_** (min)**	**Calculated mass**	**[M-H]**^**−**^ ***m/z***	**MS**^**2**^** ions,** ***m/z*** **(relative intensity)**	**Type of extract**		**Structure**
						**Acetone**	**Methanol**	
**Depsidones**								
Methylstictic acid	C_20_H_15_O_9_	11.18	399.0716	399	383, 368, 354, 325, 314, 300, 286, 256, 231, 225, 189, 94	+	+	
Gangaleoidin	C_18_H_14_Cl_2_O_7_	14.99	412.0116	412	395, 354, 326, 312, 310, 299, 164, 151, 136	-	+	
**Terpenoids**								
(-)-ent-Kauran-16α-ol (Ceruchinol)	C_20_H_33_O	11.06	289.2531	289	288, 260, 231, 182, 117	-	-	
**Fatty acids**								
Nephrosterinic acid	C_17_H_27_O_4_	14.19	295.1909	295	277 (100), 195 (21), 183 (37), 180 (9), 171 (23), 164 (11), 108 (12), 97 (14)	+	+	
Arachidonic acid	C_20_H_31_O_2_	11.80	303.2324	304	259, 244, 231, 215, 203, 189, 161, 83	+	-	
**Monocyclic aromatic**								
Atranol	C_8_H_7_O_3_	1.58	151.0395	151	87, 71, 59, 55, 43, 41	+	+	
Olivetolic acid	C_12_H_15_O_4_	12.55	223.0970	223	223, 207, 181, 163, 148, 137, 123, 121, 119, 95, 83, 79	+	-	
**Polyols**								
meso-Erythritol	C_4_H_10_O_4_	8.20	122.0579	121	121, 119, 92, 65	+	-	
**Pulvinic acid derivatives**								
Leprapinic acid	C_20_H_16_O_6_	11.97	352.094	351	288, 276, 259, 244, 231, 232, 216, 205, 158, 83, 68	+	-	
Leprapinic acid	2 M-2H + Na	8.80	725.163	725	679, 678, 677	-	+	
**Unknown compounds**								
Unknown	-	9.20	-	187	187, 169, 151, 125, 123, 97, 95, 80, 57	+	-	
2-heptyl-4,6-dimethoxy-3-methylbenzoic acid	C_17_H_26_O_4_	10.11	294.183	293	234, 250, 221, 207, 181, 178	+	-	
Trihydroxyoctadecenoic acid	C_18_H_34_O_5_	10.72	330.240	330	329, 229, 211, 201, 171, 156, 139, 127	+	-	
Unknown	-	12.00	-	360	303, 302, 301	-	+	
Unknown	-	14.38	-	390	389, 387, 371, 369, 273, 201	+	+	
Unknown	-	8.67	-	742		-	-	
Unknown	-	13.10	-	746	745, 383, 361, 235	+	-	

## Discussion

The results of the present study showed that *R. sinensis* extract significantly affected the apoptosis of OSCC cells. MTT Assay results showed that *R. sinensis* reduced the survival rate of SCC cells, and scratch test results showed that it reduced the ability of cell migration in OSCC cells. These results indicate the role of *R. sinensis* potential in apoptosis induction.

*R. sinensis* stops the cell cycle in the gap2/mitosis (G2/m) phase. This substance regulates the expression of cydin-dependent kinase1 (CDK1), cydin B1, and cydin-dependent kinase inhibitor1 (CDKN1A) genes [13]. In previous studies, the antioxidant properties of *R. sinensis* have been shown. Nazari et al. stated that *R. sinensis* and its secondary metabolites have antioxidant properties and the ability to remove toxic free radicals [24]. In addition, the anti-proliferative effects of *R. sinensis* on different cancer cell lines have been shown in previous studies. Backorova et al. has shown that secondary lichen metabolites, such as usnic acid and atranurine, induce apoptosis in HT-29 cell line by activating caspase-3 [15]. Lee et al. stated that *R. sinensis* induces apoptosis in MCF-7 and MDA-MB-231 breast cancer cells and inhibits the growth of cells in both cell groups [25].

Song-Suk-Suh et al. showed that *R. sinensis* induces apoptosis and suppresses cell growth and proliferation. It also prevents the invasion of colorectal cancer cells, and this effect is dose-dependent [26]. Nguyen et al. demonstrated the effect of the anti-cancer properties of *Flavocetraria cucullata* on various cancer cell lines [27]. Aoussar et al. conducted a study to evaluate the chemical composition and antimicrobial activity of the *Ramalina farinacea*. Their study confirmed the ability of *Ramalina farinacea* extract the inhibition of free radicals [28].

Yang et al. showed that the two compounds, including usnic acid and Potassium usnate, play a role in controlling the growth of cancer cells in in-vitro studies against various cancer cell lines. usnic acid displayed anti-cancer activity in colorectal cancer cells with limited *in-vivo* effects. usnic acid has been reported to have cytotoxic activity against HCT116, human colon cancer cells [29]. Other compounds derived from *R. sinensis* extract are protolichesterinic acid, which eliminates cancer cells by blocking the expression of heat shock protein 70 (HSP-70) in the prostate cancer cell line and activating caspase 3 in the HeLa cell line [30].

In the present study, compounds such as methystictic acid and nephrosterinic acid were present in all methanolic and acetonic extracts. A compound such as atranol was available in both acetone and methanolic extracts. Considering that methanolic and acetonic extracts had similar effects on apoptosis and necrosis of OSCC cells, it can be concluded that these main compounds have anti-cancer effects of methystictic acid and nephrosterinic acid. However, further studies are necessary to investigate the cytotoxic effect of each of these extracts alone. In addition, the acetone extract of *R. sinensis* was more effective in inhibiting cell proliferation than the methanol extract. These results are the same as those of Nguyen et al., in which acetone extract had more cytotoxic properties than ethanolic extract, attributed to the active ingredients of acetone extract of *R. sinensis*. Most of the anti-cancer effects of usnic acid on human colorectal cancer cell lines were at a concentration of 12.5–100 μM, and cell viability was assessed using the MTT assay. Their results showed that the number of invasive cells in the groups treated with usnic acid was less than other cells in the control group. Yang et al. also showed tamidoline has anti-cancer properties against colorectal cancer cells [31]. Many lichen-derived acids have acceptable anti-cancer properties, so some of these compounds, such as lecanoric acid and vulpinic acid, are much more effective than conventional drugs in reducing thyroidoxin levels [32, 33].

## Conclusions

To our knowledge, this is the first study to investigate the effects of *Ramalina sinensis* on oral squamous cell carcinoma (OSCC) cells. Our findings demonstrate *that R. sinensis* extract has a beneficial impact on OSCC cell apoptosis. These results suggest that R. sinensis extract may have anti-cancer properties and warrants further investigation into its potential mechanisms and therapeutic applications for OSCC.

### Supplementary Information

**Additional file 1.**

## Data Availability

All data generated or analysed during this study are included in this published article (and its supplementary information files).

## Abbreviations

MTT: 3-(4,5-Dimethylthiazol-2-yl)-2,5-diphenyltetrazolium bromide
CDK1: Cydin-dependent kinase1
CDKN1A: Cydin-dependent kinase inhibitor1
DMSO: Dimethyl sulfoxide
DMEM: Dulbecco's Modified Eagle Medium
FBS: Fetal bovine serum
G2/m: Gap2/mitosis
HSP-70: Heat shock protein 70
IC_50_: Inhibitory concentration at 50% inhibition
IROST: Iranian Research Organization for Science and Technology
LDB: Lichen spectral database
LC–ESI–MS/MS: Liquid chromatography positive ion electrospray ionization tandem mass spectrometry
OSCC: Oral squamous cell carcinoma
*p*: *P*-Value
PCD: Programmed cell death
PBS: Phosphate-buffered saline
PI: Propidium iodide
*R. sinensis*: *Ramalina Sinensis*
SD: Standard deviation
