# Supplementary material for: Cytotoxic, anti-proliferative, and apoptotic evaluation of Ramalina sinensis (Ascomycota, Lecanoromycetes), lichenized fungus on oral squamous cell carcinoma cell line; in-vitro study
Source: BMC Complement Med Ther. 2023 Aug 22;23:296. doi: 10.1186/s12906-023-04118-1 (PMC10463489; doi:10.1186/s12906-023-04118-1)
Supplement: Supplementary file 1 — Additional file 1. [file 12906_2023_4118_MOESM1_ESM.pdf]

NCBI Code: C152

Designation: KB

Species: Human

Tissue: mouth

Morphology: Epithelial-like

Description: The KB cell line was derived from an epidermoid carcinoma in the mouth of an adult Caucasian male. It was one of the early successful attempts to isolate and serially propagate a human cell line directly in monolayer culture on glass. The line was isolated in a medium consisting of 90% basal medium (Eagle), and 10% human serum and in the course of 350 subsequent passages has been adapted to 5% calf serum. The KB line has been used extensively in studies of cell nutrition and metabolism, cancer chemotheraphy screening, tumorigenicity and viruses. KB cells have been reported to contain human papilloma virus 18 (HPV-18) sequences.

Culture Medium: EMEM (EBSS) + 1% NEAA + 10% FBS. The cell line was adapted to RPMI 1640 + 10% FBS in NCBI.

Preservation Medium: FBS + 10% DMSO

Subculture Routine: Split confluent cultures 1:3 to 1:10, ie seeding at 1-3 x 10^4 cells/cm^2 using 0.25% trypsin or trypsin/EDTA, 5% CO2, 37°C.

Isoenzymes: LDH, G6PD, NP

Sterility: Tests for mycoplasma, bacteria and fungi were negative.

Passage No: 375

DNA Typing:

**CSF1PO:**	**FGA:**	**TH01:**	**TPOX:**	**VWA:**	**D3S1358:**	**D5S818:**	**D7S820:**	**D8S1179:**	**D13S317:**	**D16S539:**	**D18S51:**	**D21S11:**	**AMEL:**
9,10	21	7	8,12	16,18	15,18	11,12	8,12	12,13	12,14	9,10	16	27,28	X

ATCC Number: CCL-17

ECACC Number: 94050408

Reference: Proc Soc Exp Biol Med 1955, 89:362; ibid 1956, 91: 361; ibid 1957, 94:661;

Cancer Res 1958, 18:1017; Science 1961, 33:1559.

Karyology: 2n=46, near triploid to hypertriploid cell line

Chromosome Frequency Distribution (Cells /Chromosomes):

1	1	1	1	1	3	1	1	1	2	2	1	1	1	3	1	6	1	1
54	56	59	60	61	62	63	64	68	70	71	72	74	75	76	77	78	81	82

Viability: 100%, 3.5 x 10^6 cells/vial
